# De-escalated radiotherapy for HER2-overexpressing breast cancer patients with 1-3 positive lymph nodes undergoing anti-HER2 targeted therapy

**DOI:** 10.3389/fonc.2023.1280900

**Published:** 2023-11-01

**Authors:** Jing Liu, Suning Huang, Zhuofei Bi, Xiaoxue Zhang, Ziqing He, Xiaowen Lan, Yuting Tan, Xiao Lin, Wenyi Zhou, Xiaobo Huang

**Affiliations:** ^1^ Department of Radiation Oncology, Sun Yat-Sen Memorial Hospital, Sun Yat-Sen University, Guangzhou, China; ^2^ Guangdong Provincial Key Laboratory of Malignant Tumor Epigenetics and Gene Regulation, Medical Research Centre, Sun Yat-Sen Memorial Hospital, Sun Yat-Sen University, Guangzhou, China; ^3^ Yat-Sen Breast Tumor Hospital, Sun Yat-Sen Memorial Hospital, Sun Yat-Sen University, Guangzhou, China; ^4^ Department of Radiation Oncology, Guangxi Medical University Cancer Hospital, Nanning, Guangxi, China

**Keywords:** HER2 overexpression, radiotherapy, anti-Her2 targeted therapy, early breast cancer, 1-3 lymph nodes positive, regional lymph node irradiation

## Abstract

**Background:**

In the era of anti-HER2 targeted therapy, the potential clinical feasibility of considering HER2-overexpressing breast cancer cases presenting with 1-3 positive axillary lymph nodes as low-risk, and thereby contemplating postoperative radiotherapy reduction, remains an important subject for in-depth examination. The aim of this retrospective study was to evaluate the effectiveness of de-escalated radiotherapy in T1-2N1M0 HER2-overexpressing breast cancer patients receiving anti-HER2 targeted therapy. Specifically, omitting regional lymph node irradiation (RNI) after breast-conserving surgery and only performing whole-breast irradiation or omitting postmastectomy radiation therapy.

**Methods:**

A retrospective analysis was conducted on 429 patients with stage T1-2N1M0 primary invasive HER2-overexpressing breast cancer from our center between 2004 and 2018. Patients who received anti-HER2 targeted therapy were divided into an RNI group and a no RNI group to assess the role of RNI. The prognostic role of RNI was investigated via the Kaplan-Meier method and Cox proportional hazards modeling.

**Results:**

The median follow-up time was 46.8 months (range 7.1–225.8 months). In the anti-HER2 targeted therapy group RNI yielded no significant improvements in invasive disease-free survival (IDFS) (*p* = 0.940), local-regional recurrence-free survival (*p* = 0.380), distant metastases-free survival (*p* = 0.698), or overall survival (*p* = 0.403). Estrogen receptor (ER) status (hazard ratio [HR] 0.105, 95% confidence interval [CI] 0.023–0.749, *p* = 0.004) and lymph vascular invasion status (LVI) (HR 5.721, 95% CI 1.586–20.633, *p* = 0.008) were identified as independent prognostic factors for IDFS, and ER-positive and LVI-negative patients exhibited better prognoses.

**Conclusion:**

Omitting RNI may be a safe option in T1-2N1 HER2-overexpressing breast cancer patients receiving standardized anti-HER2 targeted therapy; particularly in ER-positive or LVI-negative subgroups.

## 1 Introduction

Patients with 1-3 axillary lymph node metastases and a clinical stage of N1 constitute approximately 25%–30% of early operable breast cancer cases. Radiotherapy is one of the essential components of comprehensive breast cancer treatment. The MA20 and EORTC 22922 studies (1, 2), published in 2015, demonstrated that more aggressive postoperative regional nodal irradiation (RNI) in T1-2N1 patients led to better distant metastases-free survival (DMFS), disease-free survival (DFS), or breast cancer-specific mortality (BCSM). The 20-year long-term follow-up results of the Vancouver study (3) indicated long-term overall survival (OS) benefits in the N1 subgroup of patients who received postoperative radiotherapy.

These pivotal investigations strongly endorsed postoperative radiotherapy in N1 breast cancer patients, consolidating N1 staging as a compelling and imperative indication for such therapeutic intervention, particularly in cases characterized by high clinical risk. Notably not all N1 patients benefit from postoperative radiotherapy however, and in some real-world retrospective studies improvements in local-regional recurrence (LRR) and OS derived from postoperative radiotherapy, especially RNI, have been limited; particularly in patients with relatively low clinical risk, for example, age > 40 years old, LVI-, non-triple negative breast cancer, etc. (4–8). Currently a significant proportion of clinically low-risk N1 patients, characterized by molecular subtyping, fall into the luminal breast cancer subtype, particularly luminal A type. There are concerns surrounding the potential toxic effects of RNI (9, 10), including upper limb lymphedema and brachial plexus injury leading to upper limb functional impairment, dermatitis, cardiopulmonary toxicity, and radiation pneumonitis.

HER2-overexpressing breast cancer accounts for 15%–20% of cases. In the early era without anti-HER2 targeted therapy, HER2-overexpressing breast cancer had a higher risk of recurrence and an even worse prognosis than triple-negative breast cancer, with suboptimal resistance to postoperative radiotherapy. The advent of targeted drugs in the late 1990s, specifically trastuzumab, fundamentally changed this situation however, greatly improving the treatment efficacy and prognosis of HER2-overexpressing breast cancer (11, 12). With the current standard treatment of trastuzumab and pertuzumab for N1 HER2-overexpressing breast cancer, the therapeutic effect has approached that of the best luminal A type, further weakening the value of postoperative radiotherapy for N1 breast cancer. Consequently, in the era of anti-HER2 targeted therapy, whether patients with HER2-overexpressing breast cancer with 1-3 positive axillary lymph nodes can also be considered clinically low risk and have the opportunity for reduced postoperative radiotherapy is a clinically relevant question that warrants further investigation. Nonetheless, only a limited number of reports exist in this context. In light of this we conducted a retrospective study to investigate the efficacy of de-escalating radiotherapy for T1-2N1 HER2-overexpressing breast cancer after standardized anti-HER2 targeted therapy. Specifically, omitting RNI after breast conserving surgery (BCS) and performing only whole-breast irradiation (WBI), or omitting radiotherapy after mastectomy.

## 2 Materials and methods

**Figure 1:**
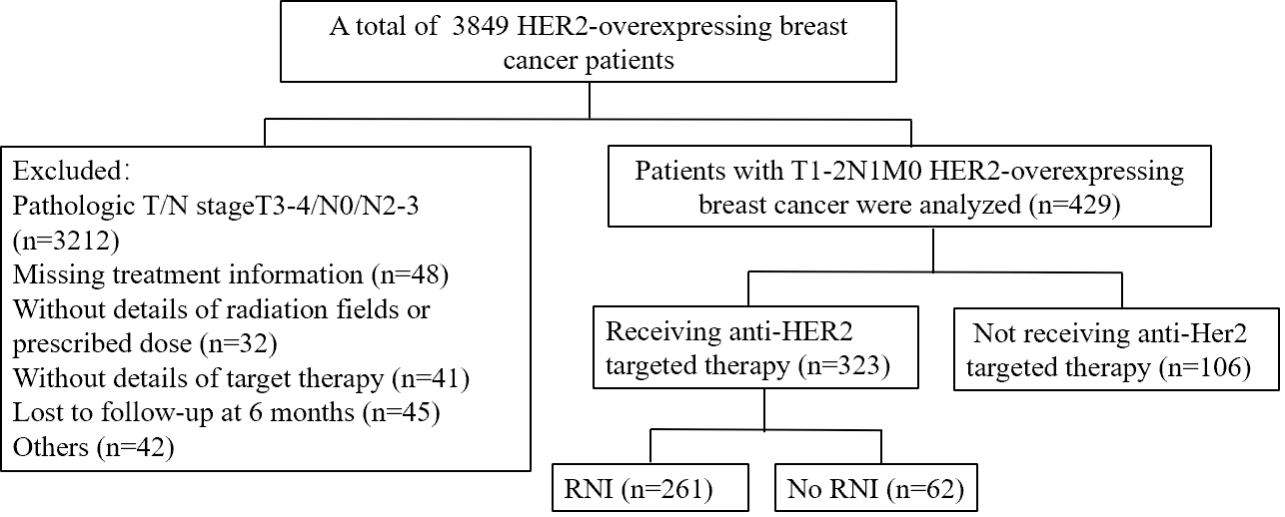
Patient screening flow chart.

A retrospective analysis of female patients diagnosed with primary invasive HER2-overexpressing breast cancer, staged T1-2N1M0 according to the 7th edition of the International Union Against Cancer/American Joint Committee on Cancer breast cancer staging system was conducted at our institution between August 2004 and April 2022. Eligible patients met the following criteria: 1) Age over 18 years; 2) pathologically confirmed HER2-overexpressing breast cancer (HER2 expression Immunohistochemistry 3+ or 1+/2+ with positive FISH); 3) presence of 1-3 positive lymph nodes with at least one pathologically confirmed, and N1mic stage; 4) T1-2 tumor stage; 5) confirmed negative surgical margins. Patients were excluded if they had pathological T3-4 or N2-3 disease, positive surgical margins, received neoadjuvant chemotherapy or other preoperative anti-HER2 targeted therapy, had clinical or pathological evidence of distant metastatic disease, had bilateral breast cancer, exhibited unclear molecular subtypes, had missing radiotherapy-related information, or were pregnant or lactating. From a total of 3,849 HER2-overexpressing patients, 429 eligible patients were included in the study. The screening process is depicted in **Figure 1**.

Patients were followed up after the treatment, and outcome data were obtained. The primary endpoint was invasive disease-free survival (IDFS). The secondary endpoints were local-regional recurrence-free survival (LRFS), DMFS, and OS. IDFS was defined as the time from surgery to the earliest occurrence of local recurrence of invasive cancer, distant metastasis, or death. LRFS was defined as the time from surgery to the earliest recurrence in the ipsilateral chest wall, breast, or regional lymph nodes, or death. DMFS was defined as the time from surgery to the earliest occurrence of distant metastasis or death. OS was defined as the time from surgery to death.

General characteristics of the patients included in the study were presented as frequencies and percentages, and were compared using Fisher’s exact test or the Chi square test. IDFS, LRFS, DMFS, and OS rates were estimated using the Kaplan-Meier method. Univariate analysis was performed using the log-rank test to identify significant independent prognostic factors. Multivariate analysis included significant factors from the univariate analysis, and hazard ratios (HRs) and 95% confidence intervals (CIs) were calculated using the Cox proportional hazards model. Two-sided *p* values < 0.05 were considered statistically significant. Statistical analyses were performed using SPSS for Windows version 26.0 (IBM Corporation, Armonk, NY, USA). The study protocol was approved by the Medical Ethics Committee of our hospital (approval number SYSKY-2023-257-01) and informed consent was waived.

## 3 Results

### 3.1 Patient characteristics and treatment

Characteristics of the entire cohort of patients are presented in **Table 1**. The median age was 50 years (range 23–85 years). All patients were diagnosed with pT1-2N1M0 HER2-overexpressing invasive breast cancer. The study population underwent either mastectomy or BCS, along with axillary lymph node dissection or sentinel lymph node biopsy, resulting in negative margins. Out of the 429 patients, 404 (94.2%) received chemotherapy. Of those 404 patients, the chemotherapy regimens included 80 on an anthracycline, cyclophosphamide, and paclitaxel regimen (19.8%), 200 on an anthracycline and cyclophosphamide regimen (49.5%), 36 on a paclitaxel and cyclophosphamide regimen (8.9%), 41 on a paclitaxel regimen (10.1%), 15 on an anthracycline, cyclophosphamide, and fluorouracil regimen (3.7%), and 32 on other regimens (8.9%). Among the entire cohort of 429 patients 351 (81.8%) were ER-positive, and 340/429 (79.3%) received endocrine therapy.

**Table 1:** Demographic and disease characteristics of the entire study cohort.

Characteristic	*n* (%)	Characteristic	*n* (%)
All	429		
Age, mean ± SD	50 ± 10	Grading	
<40	67(15.6)	G1+G2	104(24.2)
40-64	330(76.9)	G3	315(73.4)
≥65	32(7.5)	Unknown	10(2.3)
Menopausal status		With carcinoma *in situ*	
Premenopausal	232(54.1)	Yes	129(30.1)
Postmenopausal	186(43.4)	No	274(63.9)
Unknown	11(2.6)	Unknown	26(6.1)
Side		ER	
Left	211(49.2)	Positive	351(81.8)
Right	218(50.8)	Negative	78(18.2)
Operative Method		Ki67	
BCS	180(42.0)	≤15	35(8.2)
Mastectomy	249(58.0)	>15	394(91.8)
Axilla surgery		Chemotherapy	
SLNB	57(13.3)	Yes	404(94.2)
ALND	372(86.7)	No	25(5.8)
T stage		Radiotherapy	
Tis+T1	181(42.2)	RNI※	325(75.8)
T2	248(57.8)	No RNI※	104(24.2)
LVI		Endocrine therapy	
Positive	159(37.1)	Yes	340(79.3)
Negative	270(62.9)	No	89(20.7)
Pathologic stage		Target therapy	
I	15(3.5)	No	106(24.7)
II	414(96.5)	Trastu	211(49.2)
		Trastu and Pertu	112(26.1)

BCS, breast conserving surgery; SLNB, sentinel lymph node biopsy; ALND, axillary lymph node dissection; LVI, lymphatic vascular invasion; Trastu, trastuzumab; Pertu, pertuzumab; RNI, Regional nodal irradiation; RNI※, Whole-breast irradiation and RNI after BCS, and chest wall irradiation with RNI after mastectomy; No RNI※, Whole-breast irradiation after BCS, and omit irradiation after mastectomy.

A total of 323/429 (75.3%) patients received anti-HER2 targeted therapy, with 211 (49.2%) receiving trastuzumab monotherapy and 112 (26.1%) receiving dual anti-HER2 targeted therapy with trastuzumab and pertuzumab. Conversely, 106 (24.7%) patients did not receive any anti-HER2 targeted therapy. With regard to radiotherapy, 325 (75.8%) patients underwent WBI with RNI after BCS or chest wall irradiation (CWI) with RNI after mastectomy, whereas 104 (24.2%) were exempt from RNI and received only WBI after BCS, or were exempt from radiotherapy after mastectomy.

The whole breast target volume, or the chest wall target volume, and the regional lymph node target volume receive a radiation dose of 5000 cGy in 25 fractions. Radiation dose of hypofractionated radiotherapy scheme is 4000-4256 cGy in 15 to 16 fractions. For breast-conserving patients, a sequential tumor bed boost is performed after completion of whole breast irradiation. It can be delivered using conventional fractionation, with a dose of 1000 cGy in 5 fractions, or by using hypofractionation, with a dose of 798 cGy-1064 cGy in 3 to 4 fractions. Detailed comparisons of patient characteristics between the RNI and no RNI groups in the cohort that received anti-HER2 targeted therapy are presented in **Table 2**.

**Table 2:** Patient, tumor, and treatment characteristics of the group receiving anti-HER2 targeted therapy.

Factor	No RNI※	RNI※	*p* value
	Number (%)	Number (%)	
All	62	261	
Age			0.244
<40	6(9.7)	48(18.4)	
40-64	52(83.9)	200(76.6)	
≥65	4(6.5)	13(5.0)	
Menopausal status			0.813
Premenopausal	35(56.5)	151(57.9)	
Postmenopausal	25(40.3)	105(40.2)	
Unknown	2(3.2)	5(1.9)	
Operative Method			0.923
BCS	25(40.3)	107(41.0)	
Mastectomy	37(59.7)	154(59.0)	
Type of axilla surgery			0.005
SLNB	15(24.2)	28(10.7)	
ALND	47(75.8)	233(89.3)	
Side			0.789
Left	29(46.8)	127(48.7)	
Right	33(53.2)	134(51.3)	
Tumor size			0.29
≤2	30(48.4)	107(41.0)	
>2	32(51.6)	154(59.0)	
LVI			0.976
Negative	36(58.1)	151(57.9)	
Positive	26(41.9)	110(42.1)	
Pathologic stage			0.002
I	7(11.3)	5(1.9)	
II	55(88.7)	256(98.1)	
Grading			0.164
G1+G2	23(37.1)	67(25.7)	
G3	37(59.7)	188(72.0	
Unknown	2(3.2)	6(2.3)	
With carcinoma *in situ*			0.007
No	26(41.9)	152(58.2)	
Yes	34(54.8)	88(33.7)	
Unknown	2(3.2)	21(8.0)	
ER			0.432
Negative	9(14.5)	49(18.8)	
Positive	53(85.5)	212(81.2)	
Ki67			0.094
≤15	1(1.6)	23(8.8)	
>15	61(98.4)	238(91.2)	
whether endocrine therapy			0.641
No	13(21.0)	748(18.4)	
Yes	49(79.0)	213(81.6)	

BCS, breast conserving surgery; SLNB, sentinel lymph node biopsy; ALND, axillary lymph node dissection; LVI, lymphatic vascular invasion; RNI, Regional nodal irradiation; RNI※, Whole-breast irradiation and RNI after BCS, and chest wall irradiation with RNI after mastectomy; No RNI※, Whole-breast irradiation after BCS, and omit irradiation after mastectomy.

### 3.2 Survival analysis

The median follow-up time for the entire cohort was 46.8 months (range 7.1–225.8 months). During the follow-up period 22 (5.1%) patients died; 16 (3.7%) from breast cancer and 6 (1.4%) from other causes. LRR was observed in 11 (2.6%) patients, and distant metastasis occurred in 28 (6.5%) patients. The respective IDFS, LRFS, DMFS, and OS rates in the entire cohort were 90.9%, 97.4%, 93.5%, and 94.9%.

**Figure 2:**
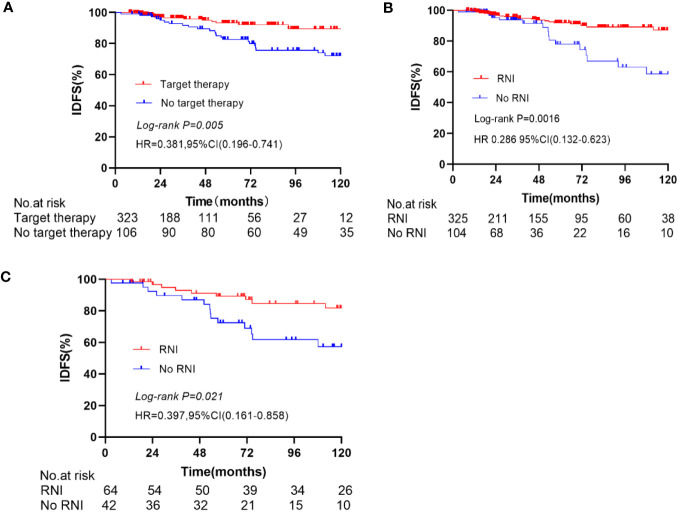
Kaplan-Meier curve for IDFS, In the entire cohort, compare the IDFS between anti-Her2 target therapy and no anti-Her2 target therapy **(A)**, RNI and No RNI **(B)**. In the no anti-Her2 targeted therapy group, compare the IDFS between RNI and no RNI **(C)**.

**Figure 3:**
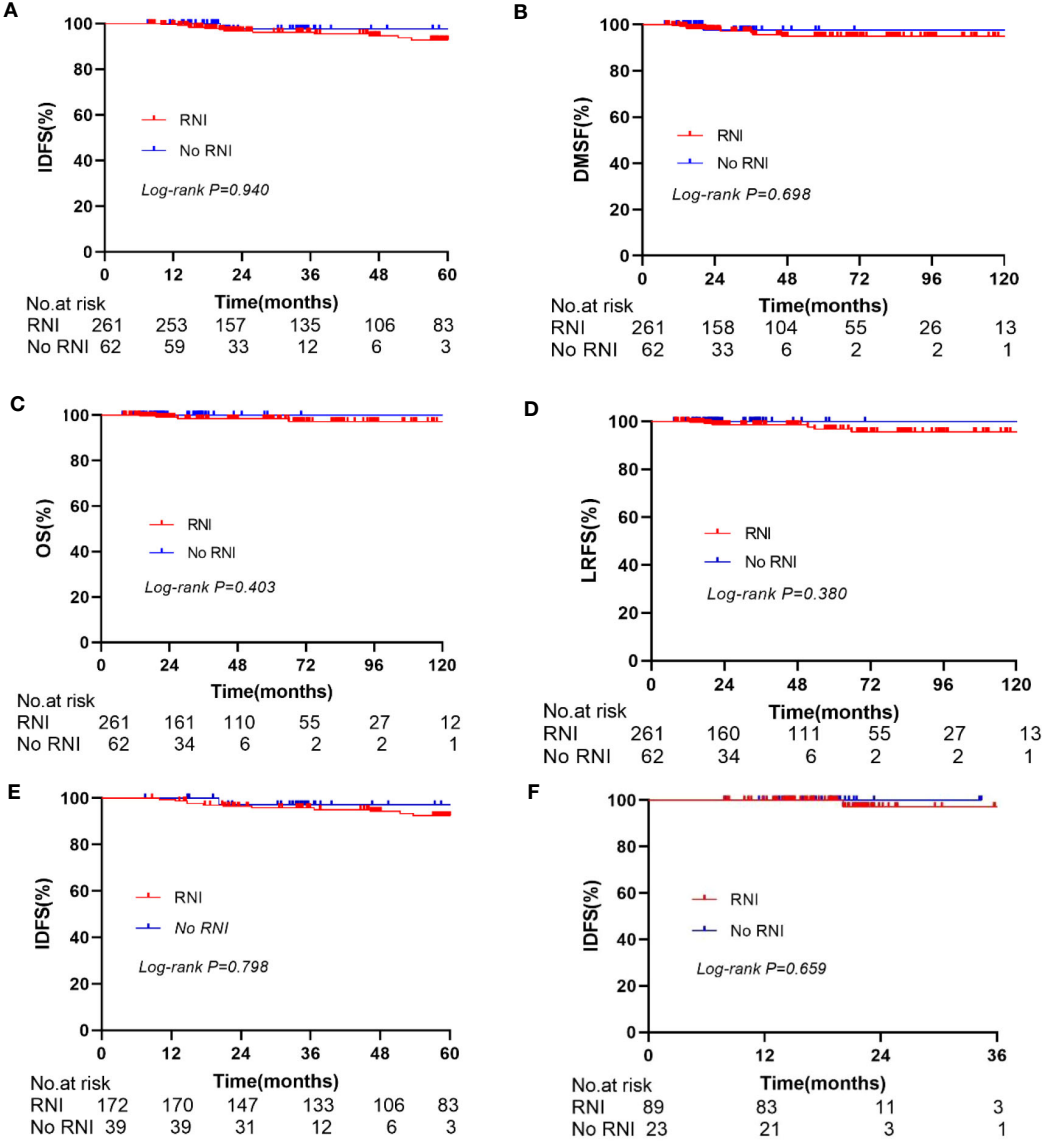
Kaplan-Meier curve for IDFS, DMFS, OS, LRFS. In the anti-Her2 cohort, compare the IDFS **(A)**, DMFS **(B)**, OS **(C)**, and LRFS **(D)** between RNI and No RNI. Compare the IDFS between RNI and No RNI in the single anti-Her2 targeted therapy group **(E)** and dual anti-Her2 targeted therapy group **(F)**.

In the entire cohort anti-HER2 targeted therapy significantly improved IDFS (HR 0.381, 95% CI 0.196–0.741, *p* = 0.005, **Figure 2A**), DMFS (HR 0.419, 95% CI 0.190–0.928, *p* = 0.032), and OS (HR 0.198, 95% CI 0.082–0.479, *p* < 0.001) compared to no anti-HER2 targeted therapy. However, no significant improvement in LRFS was observed (*p* = 0.429). Similarly, RNI was associated with significant improvements in IDFS (HR 0.286, 95% CI 0.132–0.623, *p* = 0.0016, **Figure 2B**), DMFS (HR 0.232, 95% CI 0.094–0.571, *p* = 0.002), and OS (HR 0.204, 95% CI 0.075–0.556, *p* = 0.002) compared to no RNI, but not LRFS (*p* = 0.787). In the subgroup of patients who did not undergo anti-HER2 targeted therapy, RNI was also associated with significant improvements in IDFS (HR 0.397, 95% CI 0.161–0.858, *p* = 0.021, **Figure 2C**), DMFS (HR 0.297, 95% CI 0.111–0.804, *p* = 0.017), and OS (HR 0.369, 95% CI 0.142–0.960, *p* = 0.041) compared to no RNI, but there was no significant difference in LRFS (*p* = 0.526). There were no significant differences in IDFS (*p* = 0.940, **Figure 3A**), DMFS (*p* = 0.698, **Figure 3B**), LRFS (*p* = 0.380, **Figure 3D**), or OS (*p* = 0.403, **Figure 3C**) between the RNI group and the no RNI group in the anti-HER2 targeted therapy cohort. In further analyses of single-target therapy (*p* = 0.798, **Figure 3E**) and dual-target therapy (*p* = 0.659, **Figure 3F**) there were no significant differences in IDFS between the RNI and no RNI groups. All 22 patients with N1mic disease received anti-HER2 targeted therapy, with 12 undergoing RNI and 10 not undergoing RNI. At the conclusion of the reported follow-up time no endpoint events had been observed in either group.

### 3.3 Prognostic predictors in the target therapy group

**Figure 4:**
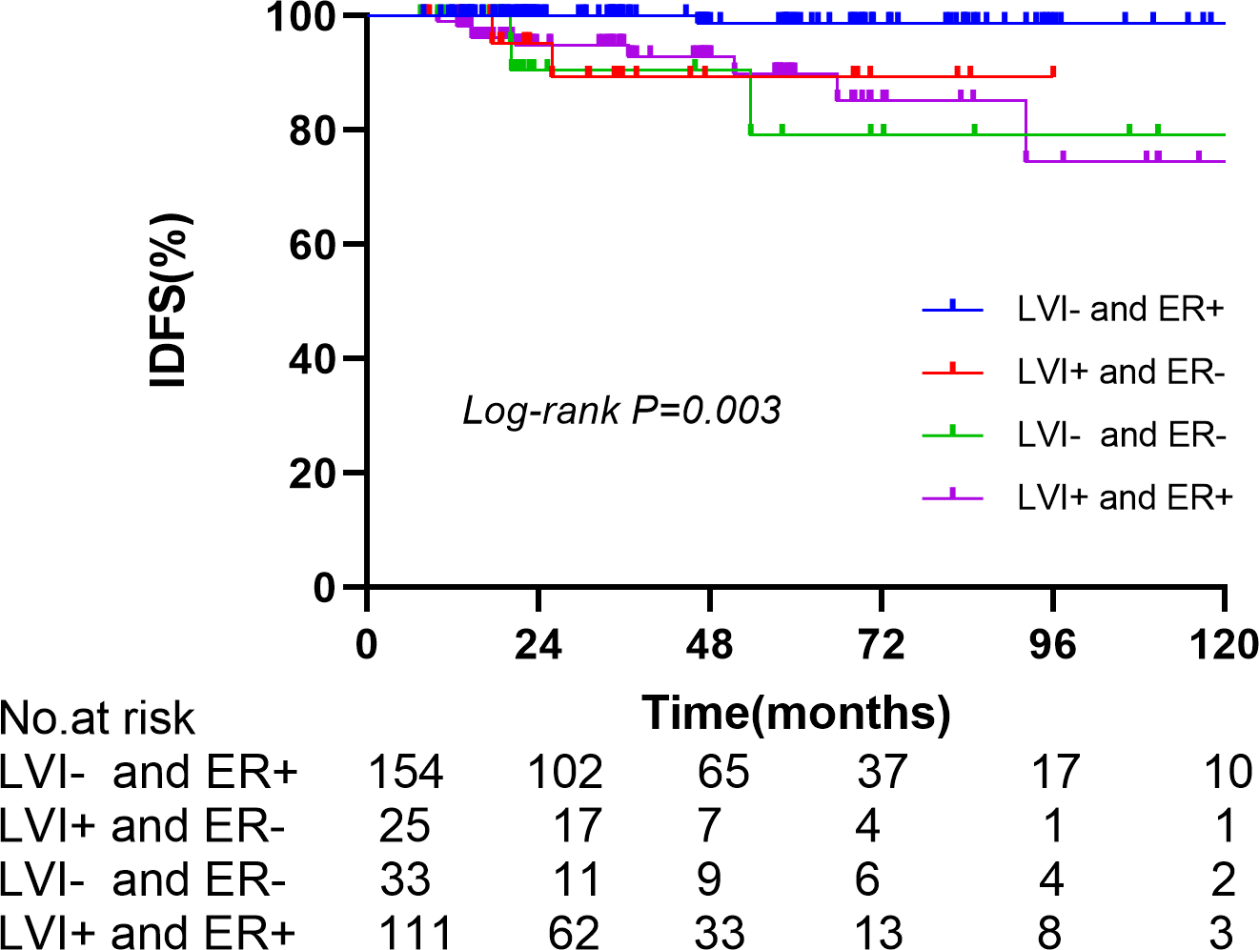
Comparison of IDFS in the anti-Her2 target therapy group by LVI and ER.

**Figure 5:**
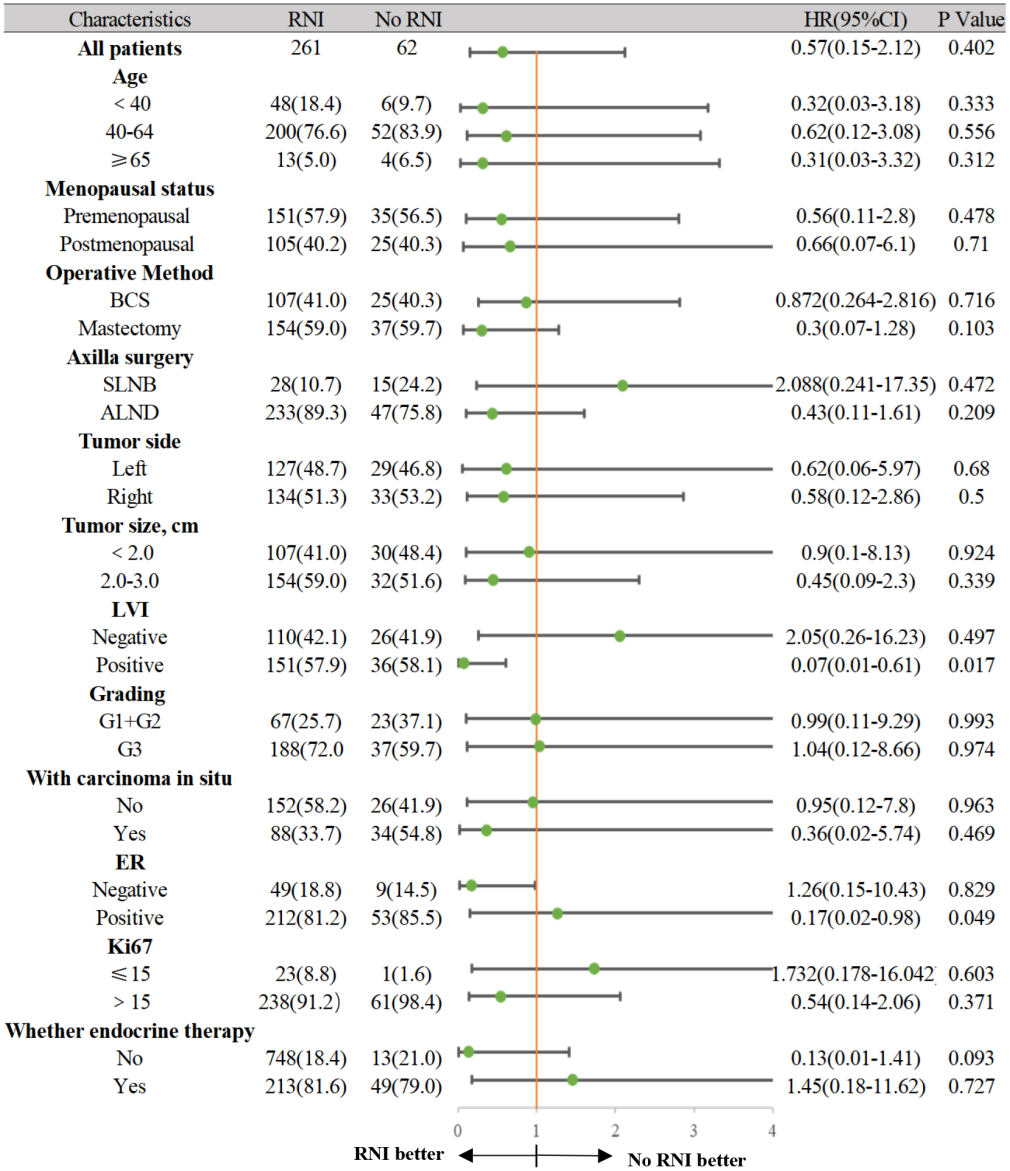
The forest plot for comparing IDFS between the RNI group and No RNI group according to different variables.

In the anti-HER2 targeted therapy group, age, menopausal status, surgical method, axillary lymph node dissection method, LVI status, tumor size, histological grade, hormone receptor status, and Ki67 levels were included in univariate analysis as prognostic factors. ER status (*p* = 0.048) and LVI status (*p* = 0.005) were significantly associated with IDFS. In multivariate analysis including predictive factors from the univariate analysis, ER status (HR 0.105, 95% CI 0.023–0.749, *p* = 0.004) and LVI status (HR 5.721, 95% CI 1.586–20.633, *p* = 0.008) were independent prognostic factors for IDFS (**Table 3**). In the group that underwent anti-HER2 targeted therapy, further investigation of associations between ER and LVI status and IDFS were conducted. Patients with ER-positive and LVI-negative status had the most favorable prognoses (*p* = 0.003) (**Figure 4**). A forest plot for comparing IDFS in the RNI group and the no RNI group according to different variables is shown in **Figure 5**. RNI treatment is advised in patients with LVI-positive or ER-negative status.

**Table 3:** Univariate and multivariate analyses of factors associated with invasive disease-free survival in patients undergoing anti-HER2 targeted therapy.

Factor	Univariate K-M	Multivariate Cox		
	*p*	HR	95% CI	*p*
Age	0.847			
<40		1		
40-64		0.329	0.021-5.22	0.431
≥65		0.304	0.027-3.37	0.332
Operative Method	0.817			
BCS		1		
Mastectomy		0.893	0.281-2.836	0.847
Type of axilla surgery	0.538			
SLNB		1		
ALND		2.099	0.252-17.460	0.493
Side	0.118			
Left		1		
Right		2.569	0.76-8.69	0.129
Tumor size	0.992			
≤2		1		
>2		1.348	0.451-4.029	0.593
LVI	0.005			
Positive		5.721	1.586-20.633	0.008
Negative		1		
ER	0.048			
Positive		0.105	0.023-0.749	0.004
Negative		1		
Ki67	0.739			
≤15		1		
>15		1.744	0.189-16.054	0.623
whether radiotherapy	0.94			
Yes		0.866	0.16-4.691	0.868
No		1		
whether endocrine therapy	0.852			
Yes		0.171	0.029-1.023	0.143
No		1		

BCS, breast conserving surgery; SLNB, sentinel lymph node biopsy; ALND, axillary lymph node dissection; LVI, lymphatic vascular invasion; RNI, Regional nodal irradiation; ER, estrogen receptor.

## 4 Discussion

The aim of the current study was to investigate the safety of de-escalating radiotherapy for T1-2N1 HER2-overexpressing breast cancer after standard anti-HER2 targeted therapy. Specifically, we evaluated the safety of omitting RNI after BCS with only WBI and no radiotherapy after mastectomy. In the anti-HER2 targeted therapy group, comparisons between RNI and no RNI subgroups did not indicate any improvements in IDFS, DMFS, OS, or LRFS. In further analysis of single-target therapy IDFS was not improved regardless of whether RNI was used or not. For dual-target therapy, IDFS was not improved with or without RNI either. These findings suggest that the benefits of RNI may be limited in the context of anti-HER2 targeted therapy.

The study by Shirin Muhsen et al. indicates that T1-2N1 patients avoided PMRT and maintained a low LRR (7%). In patients who did not receive PMRT, the presence of age over 40 and LVI showed a significant correlation with LRR (5), similar to our research findings. In the HERA trial (13), Patients with 1 to 3 positive lymph nodes had LRR-free survival of 97% in the PMRT group compared with 90% in the no PMRT group (HR 0.28, *p* = 0.004). Our results may differ because our study included a higher proportion of ER-positive breast cancer patients (81.8%). This subgroup of patients receiving both anti-HER2 targeted therapy and endocrine therapy exhibit more favorable biological behavior with a lower risk of LRR, resulting in limited benefit from radiotherapy. In the HERA trial the proportion of such patients was only 50.0%. Moreover, we included relatively low-risk T1-2 patients and did not include T3 patients. The HERA study included T1-3 patients. Lastly, our analysis included approximately 40% of patients who underwent BCS and received WBI, with the omission of RNI. WBI after BCS can further enhance the activated immune microenvironment, augmenting tumor cell sensitivity to T cell-mediated anti-tumor effects, and facilitating cancer cell recognition and elimination. Consequently, this subset of patients exhibits a lower LRR risk. However, all patients included in the HERA trial had undergone mastectomy. Furthermore, the HERA trial indicates that for lymph node-negative (N0) patients who receive anti HER2-targeted therapy, there was no significant difference in the rate of locoregional recurrence following post-mastectomy radiation therapy (PMRT) (P-value = 0.96).

In the entire cohort in the present study, anti-HER2 targeted therapy improved IDFS, DMFS, and OS compared to without anti-HER2 targeted therapy, but did not improve LRFS. These observations are consistent with previous research indicating that anti-HER2 targeted therapy greatly improves the prognosis and outcomes of HER2-overexpressing breast cancer (11, 12, 14). The significant improvement of IDFS, DMFS, and OS with anti-HER2 targeted therapy confirms the transformative significance of these treatments for this breast cancer subtype (15, 16). In the non-anti-HER2 targeted therapy group, RNI was associated with improved IDFS, DMFS, and OS, but not LRFS. A possible reason for this in this subset of patients is the elevated risk of distant metastasis. This finding is consistent with results from the MA20 and EORTC22922 studies (1, 2). Radiotherapy can induce an abscopal effect by activating an anti-tumor immune response (17–19). Following radiation exposure, tumor cells undergo DNA double-strand breaks and cell membrane damage, leading to the rapid release of tumor-associated antigens into the bloodstream. This in turn triggers the release of danger-associated molecular patterns, collectively recruiting and activating the immunological functions of natural killer cells and dendritic cells to mount an immune attack against tumor cells. This process reduces the risk of distant tumor metastasis, and yields survival benefits. The lack of improvement in LRFS may be due to a low-risk profile in the overall cohort of HER2-positive N1 breast cancer patients, and a limited number of positive recurrence cases.

In recent studies the outcomes of N1 disease in breast cancer patients have exhibited improvement, primarily reflecting advances in cancer screening, surgical techniques, and systemic therapies (20–22). In the initial trials of postoperative radiotherapy, adjuvant systemic therapies included CMF chemotherapy for premenopausal women, and tamoxifen for postmenopausal women. Over the past two decades, more effective adjuvant systemic therapies and improved surgical techniques have led to advances in local and distant metastasis control. The benefits of postoperative radiotherapy, particularly RNI have diminished.

With improvements in axillary staging, the advent of modern chemotherapy regimens, the use of aromatase inhibitors, and the implementation of targeted therapies the absolute risk of breast cancer recurrence has significantly decreased. In modern series the 5-year LRR rates in patients without postoperative radiotherapy range from 3.2% to 6.1%, and even after 10 years of follow-up they remain below 10.0% (13, 20, 23–25). The results of the current study are concordant with this. Recent studies have reported LRR rates of 0.26% to 1.70% for HER2-positive patients treated with modern approaches (26, 27). Particularly noteworthy is the pivotal release of the APHINITY study (28), which demonstrates that dual targeting mildly reduces the risk of recurrence further, and improves OS. Therefore, the absolute benefits of RNI in patients with T1-2N1 disease may actually be minimal, as any survival benefits of RNI may be offset by its side effects. In the future a greater number of HER2+ patients will be optimized to receive novel neoadjuvant treatment modalities, enabling better differentiation between those who achieve pathological complete response and those who do not (29, 30). This distinction will aid in determining whether patients belong to trastuzumab or pertuzumab sensitive or resistant subtypes. In sensitive patients, targeted therapy can effectively reduce the risk of recurrence and improve overall treatment efficacy and prognosis, potentially allowing for a reduction in radiotherapy. Conversely, non-sensitive patients with poorer prognoses and a higher risk of LRR will require further optimization and intensification of postoperative radiotherapy, building upon secondary targeted therapies such as T-DM1 or small molecule TKIs, and refining RNI (31–33). We eagerly anticipate the final results of the NSABP B51/RTOG1304 Phase III clinical trial (34). This trial was designed to assess whether RNI improves DFS in breast cancer patients with cT1~3N1 disease who achieve ypN0 status following neoadjuvant chemotherapy and surgery. This trial is poised to answer the critical question of whether, by omitting postoperative radiation therapy, it is possible to proactively employ the pathological response rates in the breast or lymph nodes to identify a subgroup of patients at low risk of recurrence.

Multivariate analysis of anti-HER2 targeted therapy subgroups indicated that ER and LVI status were independent prognostic factors for IDFS. Patients with ER-positive and LVI-negative status had the best prognosis. Patients with positive N1 hormone receptors had a higher rate of local-regional control after PMRT (35). Those results are consistent with subtype analysis in the Danish trial, which reported decreased local-regional control benefits of PMRT in ER or PR-negative HER-2-positive subtypes (36–38). Biologically, this can be explained as radiation sensitivity conferred by the ER signal, which accelerates the G1/S phase transition and reduces DNA repair (39, 40). Therefore, in patients with negative hormone receptor status, dose escalation or radiation sensitization methods are reasonable future considerations (41). Furthermore, LVI-positive disease progresses faster. Therefore, more aggressive treatment strategies may be needed for this subgroup of patients, including closer follow-up and observation to take appropriate measures promptly if there are signs of recurrence. Based on this, in breast cancer patients with 1-3 positive lymph nodes the paramount objective is to identify the high-risk individuals hidden within the clinically low-risk patient cohort who would benefit from postoperative radiotherapy, while sparing those who are truly low-risk from unnecessary radiation therapy. This calls for the effective implementation of precise molecular genetic predictive models. To address this clinical issue two registered clinical studies are currently underway, the RIGAIN trial based on the 28-gene model and the Tailor RT study based on the 21-gene model. The 28-gene model stands as the sole gene-based predictive model capable of forecasting recurrence in HER2-overexpressing breast cancer. We eagerly await the release of the final results.

The limitations of the present study include its retrospective nature and potential selection bias, as well as the relatively small sample size and limited follow-up time with respect to certain analyses. The study population was also derived from a single center, which may limit the generalizability of the results. Nonetheless, the results of the study contribute to an increasing body of evidence supporting the use of anti-HER2 targeted therapy for treating HER2-overexpressing breast cancer, and underscore the importance of considering patient-specific factors when determining optimal treatment strategies.

With regard to future research directions, investigating larger sample sizes across multiple centers is crucial, to more accurately assess the safety of omitting RNI in both ER-positive and HER2-overexpressing breast cancer patients with 1-3 positive lymph nodes who are receiving targeted therapy. The roles of potentially influential factors such as tumor staging and gene expression profiles in breast cancer treatment should be further investigated, to provide more comprehensive treatment recommendations. Based on the application of accurate predictive recurrence models to assess risks of recurrence, individualized treatment approaches such as the 28-gene assay should be implemented. Lastly, the effects of novel anti-HER2 drugs on breast cancer prognoses should be further investigated, to provide patients with the latest treatment options.

## 5 Conclusion

This study revealed that in the era of anti-HER2 targeted therapy, HER2-overexpressing breast cancer with 1-3 positive axillary lymph nodes and positive hormone receptor status has gradually evolved into a clinically low-risk subtype. Postoperative RNI did not confer additional benefits to these patients, suggesting that exemption from RNI may be a safe approach. Additionally, ER status and LVI status had prognostic significance and should be thoroughly considered in treatment decisions. Nonetheless, individualized assessment of the appropriateness of RNI remains essential. Future research endeavors should encompass larger sample sizes and multi-center collaborations to provide more precise treatment recommendations in this patient population.

## Data availability statement

The raw data supporting the conclusions of this article will be made available by the authors, without undue reservation.

## Ethics statement

The studies involving humans were approved by the Institutional Review Board at Sun Yat-Sen Memorial Hospital, Sun Yat-Sen University (approval number SYSKY-2023-257-01). The studies were conducted in accordance with the local legislation and institutional requirements. Written informed consent for participation was not required from the participants or the participants’ legal guardians/next of kin in accordance with the national legislation and institutional requirements.

## Author contributions

JL: Conceptualization, Data curation, Investigation, Methodology, Project administration, Writing – original draft, Writing – review & editing. SH: Methodology, Supervision, Writing – review & editing. ZB: Data curation, Validation, Writing – review & editing. XZ: Data curation, Supervision, Validation, Writing – review & editing. ZH: Data curation, Validation, Writing – review & editing. XLan: Data curation, Writing – review & editing. YT: Data curation, Validation, Writing – review & editing. XLin: Data curation, Validation, Writing – review & editing. WZ: Data curation, Writing – review & editing. XH: Conceptualization, Formal Analysis, Funding acquisition, Methodology, Supervision, Writing – review & editing.
